# Sense of Coherence and Mental Health in College Students After Returning to School During COVID-19: The Moderating Role of Media Exposure

**DOI:** 10.3389/fpsyg.2021.687928

**Published:** 2021-07-22

**Authors:** Man Li, Zhansheng Xu, Xinyue He, Jiahui Zhang, Rui Song, Wenjin Duan, Tour Liu, Haibo Yang

**Affiliations:** ^1^Key Research Base of Humanities and Social Sciences of the Ministry of Education, Tianjin Normal University, Academy of Psychology and Behavior, Tianjin, China; ^2^Faculty of Psychology, Tianjin Normal University, Tianjin, China; ^3^Tianjin Social Science Laboratory of Students’ Mental Development and Learning, Tianjin, China

**Keywords:** sense of coherence, mental health, COVID-19, media exposure, anxiety, depression

## Abstract

The COVID-19 pandemic not only threatens people’s physical health, but also affects their mental health in the long term. Although people had returned to work and school, they are closely monitoring the development of the epidemic and taking preventive measures. This study attempted to examine the relationship between media exposure, sense of coherence (SOC) and mental health, and the moderating effect of media exposure in college students after returning to school. In the present study, we conducted a cross sectional survey on 424 college students returning to school around May 2020. Self-report questionnaires were used to assess media exposure scale, SOC, depression, anxiety and stress. Correlation and moderation analysis was conducted. The results showed that (1) negative epidemic information exposure, rather than positive epidemic information exposure, was significantly associated with depression, anxiety, and stress. (2) SOC was also associated with depression, anxiety, and stress. (3) The effect of SOC on depression was modified by negative epidemic information exposure. With the increase of negative epidemic information exposure, the predictive effect of SOC on depression is increasing gradually. These findings demonstrated that negative epidemic information exposure was associated with an increased psychological distress in the sample. A high SOC played a certain protective role in the adaptation of college students in the post-epidemic period. It is important to find more ways to increase the colleges’ SOC level and avoid negative information exposure.

## Introduction

According to the Worldometers, as of January 2021, the number of COVID-19 patients has exceeded 91.35 million in worldwide. As an international public health emergency, the COVID-19 pandemic has had serious social, psychological, and economic impacts on a global scale (Li et al., 2020; Yang et al., 2020). Recent studies have shown that anxiety and depression symptoms are more common in the population during COVID-19 (Tng et al., 2020). Salari et al. (2020) conducted a meta-analysis about the impact of COVID-19 on mental health in the general population prior to May 2020 and found that the prevalence of stress was 29.6% with a total sample size of 9074, the prevalence of anxiety 31.9% with a sample size of 63,439 and the prevalence of depression 33.7% with a total sample size of 44,531 people.

The negative emotions caused by COVID-19 may further damage the physical and mental health of individuals (Ren et al., 2020). Previous studies showed that the individual’s resilience will decline under long-term chronic stresses, and then do harm to individuals’ physical and mental health (Bijlsma and Loeschcke, 2005; De Kloet et al., 2005). A recent web-based cross-sectional study showed that during the COVID-19 outbreak, the prevalence of anxiety and depressive symptoms in young people was significantly higher than that in older people (Huang and Zhao, 2020). Depression is significantly correlated with maladjustment in college students (Horgan et al., 2016). As the epidemic has been brought under control, some college students have returned to school. Therefore, the research on depression, anxiety and stress of college students is of certain practical significance to understand the current psychological state of college students and provide guidance for college students to better adapt to their study and life in the post-epidemic period.

Sense of coherence (SOC) is a stable psychological tendency of the individual’s overall feeling and cognition of life, and it is a universal, lasting, and dynamic self-confidence within the individual. SOC consists of three factors: (1) in the course of life, internal and external pressures from individuals are structured, predictable and explainable (understandable); (2) individuals have access to resources to deal with these stresses (controllable); (3) these stresses are challenging and worthy of investment and participation (sense of meaning) (Antonovsky, 1987, p. 191). According to the salutogenic model, it is more important to pay attention to people’s access to health resources and the process of health promotion than to risk factors (Antonovsky, 1979). As the core concept of beneficial health model theory, SOC is a kind of ability to successfully cope with stress (Antonovsky, 1993), which aims to explain the reasons for individual differences in stress situations (Geyer, 1997). Previous studies have shown that there is a close relationship between SOC and mental health (Togari et al., 2008), which plays an important intermediary or buffer role between stressful life events and emotional symptoms (such as depression, anxiety) (Schnyder et al., 1999).

During the COVID-19 pandemic, a long-term and strict quarantine policy enabled young people to use mobile media more to get information. Some studies have shown that watching negative epidemic reports (such as epidemic severity, hospital reports) is associated with more depression, while watching positive epidemic reports (such as heroic behavior, expert speeches, etc.) is associated with less depression (Chao et al., 2020). However, the spread of COVID-19 news in the mainstream media is dominated by negative epidemic information (Cowper, 2020; Dyer, 2020; Wang Y. et al., 2020), and more studies have shown that frequent contact with COVID-19 news in the mainstream media is associated with higher levels of audience depression (Keles et al., 2020; Olagoke et al., 2020). Social media is also one of the important channels to update COVID-19’s information, but social media networks may involve a lot of false information, thus exacerbating public panic (Kilgo et al., 2019). In addition, social media is rife with negative emotions from the epidemic, which can infect the social network (Kramer et al., 2014), and excessive use of social media can also increase the risk of depression (Hunt et al., 2018). Bendau et al. (2020) found that the frequency, duration, and diversity of media exposure were significantly positively correlated with depression. For example, the longer people spent on social media, the more severe their depressive symptoms were (Lee et al., 2020). Therefore, further research on media exposure is helpful for us to explore how to interfere with negative emotions.

**FIGURE 1:**
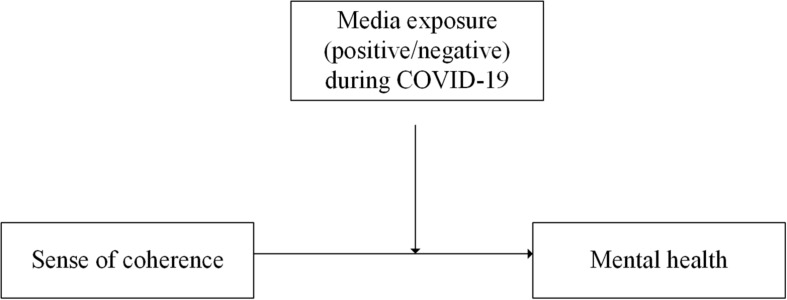
Hypothetical model of the moderating effect for the relationship between sense of coherence (SOC) and mental health.

In the post-epidemic era, students return to campus one after another, but what is the emotional adaptation of college students after the epidemic pressure and long-term home isolation? The influencing factors of college students’ adaptation after returning to school are worth discussing. Previous studies have shown that SOC is a protective factor of mental health (Togari et al., 2008), and there is a positive correlation between media exposure and individual psychological abnormalities (Bendau et al., 2020). In view of the relationship between SOC, media exposure and mental health, we can infer that higher SOC and less media exposure may be related to fewer negative emotions. However, few studies have examined the regulatory effect of media exposure on the relationship between psychological identity and emotion. The Antonovsky’s salutogenic model showed that the formation of SOC is mainly influenced by two kinds of factors, one is generalized resistance deficiency and the other is generalized resistance resources. The stress appraisal theory showed that two cognitive processes were related to coping stress: the primary appraisal and secondary appraisal (Hjemdal et al., 2006). The primary appraisal includes determining whether the event is stressful and whether the stressor poses a threat or causes harm. The secondary appraisal mainly includes the individual’s assessment of the resources. By comparing the salutogenic model and the stress appraisal theory, it can be found that the content of primary appraisal is highly similar to the generalized resistance deficiency, and the content of secondary appraisal is also very similar to the generalized resistance resources. Studies have shown that media exposure related to COVID-19 is associated with more obvious psychological stress (Bendau et al., 2020), and media exposure may affect individuals’ perception of stress. So, we reason that media exposure may affect the development of college students’ SOC through stress evaluation, and then play a moderating role in the relationship between SOC and mental health. In addition, since positive epidemic information exposure and negative epidemic information exposure may cause individuals to make opposite stress evaluations of COVID-19, there may be differences in their effects on SOC and mental health. Therefore, this study subdivides media exposure into positive epidemic information exposure and negative epidemic information exposure, in order to explore the relationship between psychological identity, media exposure, and college students’ emotional adaptation after returning to school, and further investigate the moderating effect of positive and negative epidemic information exposure on psychological identity and emotional adaptation of college students (see Figure 1). This study hypothesizes that media exposure plays a moderating role in the relationship between SOC and mental health, and that the effects of positive and negative epidemic information exposure are different.

## Materials and Methods

### Study Design and Procedure

A cross-sectional study was conducted on college students returning to school around May 2020. Self-report questionnaires were used to assess media exposure scale, SOC, depression, anxiety and stress. SPSS24.0 was used for statistical analysis of the questionnaire results. By the end of April 2020, China’s epidemic prevention and control efforts had entered a normal stage. Students began to return to campus in May, 2020. The current study was conducted 1 month after their returning, from June 2nd, 2020 to June 12th, 2020. We distributed QR codes of questionnaires in the classrooms and libraries of Tianjin Normal University. In addition, we also released questionnaires via WeChat and QQ, two popular social media platforms in China. The study purpose was disclosed and the consent to participate was provided. This study was approved by the ethical committee of Tianjin Normal University.

### Participants

A total of 500 participants were recruited. Subjects were excluded if they filled out the questionnaire too quickly (less than 60 s, *n* = 60) and filled with obvious repetition answers (*n* = 16). As such, 424 college students (*M* = 20.49 years, *SD* = 1.95) were included in our study. Previous literature indicated that the observations (i.e., the collected questionnaires) should be 5–10 times the number of items for considered variables (Austin and Steyerberg, 2015). Because 44 items were used to assess our considered variables in the present study, the minimum sample size should be 220–440. The sample size (*n* = 424) was adequate for the conducted statistical analyses.

### Measures

#### Epidemic-Related Information Exposure Through Media

Ten items following previous research (Hall et al., 2019) were used to examine participants’ media exposure during the COVID-19 pandemic. Five questions asked about the positive epidemic information that participants viewed, including positive responses to the epidemic, stories about heroes, official reports and interviews about the epidemic, good news about patients being discharged from hospitals, and encouraging videos or songs related to the epidemic (range from 1 = Almost nothing to 4 = Very much). Five questions asked about the negative epidemic information that participants viewed, including the lack of treatment for COVID-19 patients, the helplessness and suffering of people in affected areas, poor preparedness, the increasing number of COVID-19 diagnoses and deaths, and the lack of medical supplies (range from 1 = Almost nothing to 4 = Very much). The scores of each part were calculated by summing the scores of relevant items, ranging from 5 to 20 points. The higher the score, the higher the media exposure.

#### Sense of Coherence Scale (SOC-13)

The validated Chinese version of the Sense of Coherence Scale-13 (SOC-13) was used in our study (Antonovsky, 1993; Bao and Liu, 2005). The items address the degree to which participants experience various aspects of life as meaningful, comprehensible and manageable. This version consists of 13 items rated on 7-point scales with the anchors defined. The total scale score ranges from 13 to 91, with higher scores denoting a stronger SOC. The Cronbach’s alpha coefficient is reported to be 0.76 (Bao and Liu, 2005).

#### Depression-Anxiety-Stress Scale (DASS-21)

We used the validated Chinese version of the 21-item Depression Anxiety Stress Scale (DASS-21) (Gong et al., 2010) to measure depression, anxiety, and stress symptoms. This scale is comprised of three subscales assessing anxiety (7 items), depression (7 items), and stress (7 items). Respondents are asked to respond on a Likert scale from 0 (did not apply to me at all) to 3 (applied to me very much or most of the time). Each subscale score ranges from 0–21, with higher scores indicating higher degrees of anxiety, depression or stress. As the DASS-21 is a short form version of the DASS (42 items), the final score of each scale were multiplied by two, so that they can be compared with the normal DASS scores (Lovibond and Lovibond, 1995; Antony et al., 1998). Example items include “I felt I was close to panic” for anxiety, “I couldn’t seem to experience any positive feeling at all” for depression, and “I found it difficult to relax” for stress. In the current study, the time frame adopted was “during the last week.” The internal consistencies for each scale for DASS-21 in the current study were as follows: depression, 0.77; anxiety, 0.79; stress, 0.76.

### Statistical Analysis

SPSS 24.0 was used for descriptive statistics and correlation analysis of the data, and then Model 1 (as a simple regulation model) in SPSS Process component developed by Hayes (2013) was used to test the regulation effect, and the regulation effect was analyzed by Bootstrap method. Hierarchical multiple regression was used to investigate the predictive effect of positive and negative epidemic information exposure on depression, anxiety and stress, and their moderating effect on the relationship between SOC and depression, anxiety and stress after controlling for age and gender. The subjects were divided into low group (Z – 1SD) and high group (Z + 1SD) according to the standard score of exposure to negative epidemic information. Participants whose scores were more than one standard deviation above the mean were classified as high group, and those whose scores were less than one standard deviation below the mean were classified as low group. The simple slope test was used to further investigate the influence of SOC on depression at different levels of exposure to negative epidemic information.

## Results

### Single Factor Common Deviation Test Analyses

According to Harman single factor common deviation test, exploratory factor analysis was conducted for six factors: negative epidemic information exposure, positive epidemic information exposure, SOC, depression, anxiety, and stress. The first factor explained 27.93% of variation, less than 40%, which meant there was no common deviation in our data.

### Descriptive Analyses

Among the participants, 116 were male (27.4%) and 308 were female (72.6%). The participants were mainly from Tianjin Normal University, with a small number of participants from other colleges. For the regional distribution, 325 (76.65%) students’ college was in Tianjin, 55 (12.97%) in Guangxi, 9 (2.12%) in Beijing, 5 (1.18%) in Zhejiang, 5 (1.18%) in Guizhou, and 20 (4.72%) in other regions (Jiangsu, Shanxi, Chongqing and other 13 regions). During the COVID-19 pandemic, 87 students stayed in Tianjin (20.52%), 79 in Guangxi (18.63%), 33 in Shanxi (7.78%), 30 in Hebei (7.08%), 23 in Henan (5.42%), and 154 (36.32%) in other regions (Sichuan, Jiangxi, Guizhou and other 22 regions). 18 participants (4.25%) did not specify their location during the COVID-19 pandemic. Among them, 171 freshmen (40.3%), 108 sophomores (25.5%), 75 juniors (17.7%), 30 seniors (7.1%), and 40 postgraduates or above (9.4%). There are 232 (54.7%) students majoring in science and engineering, 137 (32.3%) students majoring in liberal arts (philosophy, law, etc.), 22 (5.2%) students majoring in art (PE, music, etc.) and 33 (7.8%) students majoring in other subjects.

**FIGURE 2:**
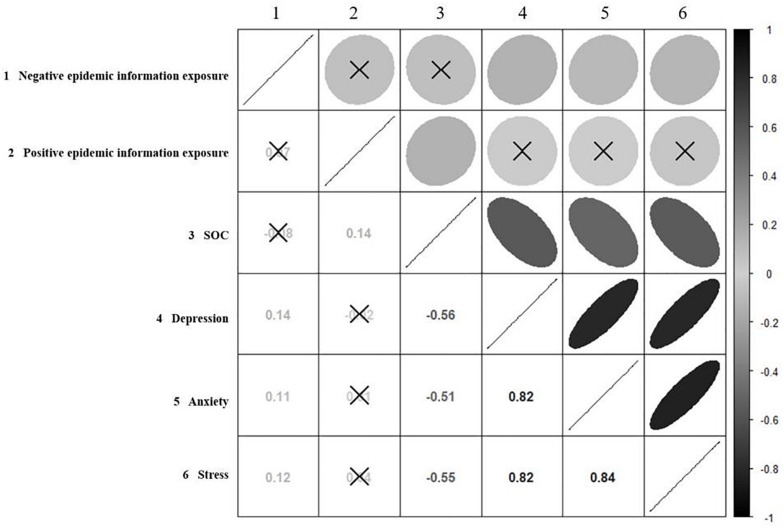
Correlation matrix of variables used in the present study. The correlation coefficients for each pair of variables were shown with the numerical values and ellipses in the matrix. The gray scale indicates the correlation coefficients. Black crosses indicate that the correlation was not significant (*P* > 0.05).

Table 1 shows the descriptive statistics and correlations between media information exposure, SOC, depression, anxiety, and stress symptoms in the sample. Most participants read a lot of positive news through the media and a relatively little negative news. The average data showed that the students had a related lower level of depression, anxiety, and stress after exposed to multiple media information related to COVID-19. Gender was positively correlated with positive epidemic exposure (*r* = 0.10, *p* < 0.05; 1 = male, 2 = female), indicating that female reported more positive epidemic exposure. Age was positively correlated with negative epidemic information exposure (*r* = 0.14, *p* < 0.01), which means that the older people reported more negative epidemic exposure than younger people. In Figure 2, the results showed that negative epidemic information exposure was significantly positively correlated with depression (*r* = 0.14, *p* < 0.01), anxiety (*r* = 0.11, *p* < 0.05), and stress (*r* = 0.12, *p* < 0.05). Positive epidemic information exposure was not significantly correlated with depression (*r* = –0.02, *p* = 0.68), anxiety (*r* = 0.01, *p* = 0.84), and stress (*r* = 0.04, *p* = 0.47). SOC was negatively correlated with depression (*r* = –0.56, *p* < 0.01), anxiety (*r* = –0.51, *p* < 0.01), and stress (*r* = –0.55, *p* < 0.01), and positively correlated with positive epidemic information exposure (*r* = 0.14, *p* < 0.01). There was no significant correlation between negative epidemic information exposure and SOC (*r* = –0.08, *p* = 0.09) or positive epidemic information exposure (*r* = 0.07, *p* = 0.14). The result of correlation analysis conforms to the condition of regulating effect test and is suitable for further analysis.

**TABLE 1:** Descriptive statistics and correlations among study variables (*N* = 424).

	**M**	**SD**	**1**	**2**	**3**	**4**	**5**	**6**	**7**	**8**	**9**
1. Gender											
2. Course			0.12*								
3. School location			−0.37**	−0.13**							
4. Age	20.49	1.95	–0.07	0.08	–0.07						
5. Negative epidemic information exposure	9.98	3.09	0.09	0.05	−0.12*	0.14**					
6. Positive epidemic information exposure	16.17	2.96	0.10*	0.06	–0.08	–0.05	0.07				
7. SOC	55.39	10.56	–0.07	–0.07	0.07	0.04	–0.08	0.14**			
8. Depression	6.94	7.7	–0.01	0.04	–0.04	–0.03	0.14**	–0.02	−0.56**		
9. Anxiety	7.62	7.38	–0.01	0.05	–0.02	–0.06	0.11*	0.01	−0.51**	0.82**	
10. Stress	9.01	8.02	–0.04	0.03	–0.01	0.02	0.12*	0.04	−0.55**	0.82**	0.84**

Gender: 1 = male, 2 = female; Course:1 = science and engineering, 2 = liberal arts, 3 = art, 4 = other subjects; School location: 1 = Tianjin, 2 = Guangxi, 3 = other regions; SOC: sense of coherence; *p < 0.05; **p < 0.01.

### Moderation Effect Analyses

The models containing depression, anxiety, and stress were established, respectively. Since gender and age were significantly correlated with positive epidemic information exposure and negative epidemic information exposure, respectively, the moderating effect of gender and as control variables on positive and negative epidemic information exposure when the models were tested. The moderation effect of positive epidemic information exposure and negative epidemic information exposure between SOC and depression was tested, respectively. And the same procedure was used to test the effect on anxiety and stress.

Table 2 presents the results of regression analysis, the product of SOC and positive epidemic information exposure had no significant predictive effect on depression (β = 0.01, *p* = 0.42, 95% CI [–0.01, 0.03]), anxiety (β = 0.01, *p* = 0.50, 95% CI [–0.01, 0.03]), and stress (β = 0.004, *p* = 0.71, 95% CI [–0.02, 0.02]). It indicates that positive epidemic information exposure has no moderating effect between SOC and depression, anxiety, and stress. In addition, the product of SOC and negative epidemic information exposure had a significant negative predictive effect on depression (β = –0.03, *p* < 0.01, 95% CI [–0.04, –0.01]) (Table 3), indicating that exposure to negative epidemic information can regulate the effect of SOC on depression. The product of SOC and negative epidemic information exposure had no significant predictive effect on anxiety (β = –0.01, *p* = 0.16, 95% CI [–0.03, 0.01]) and stress (β = –0.02, *p* = –0.11, 95% CI [–0.04, 0.004]). It indicates that negative epidemic information exposure has no moderating effect on the influence of SOC on anxiety and stress.

**TABLE 2:** Hierarchical regression analyses of positive epidemic information exposure on negative emotions (*N* = 424).

	**Depression**				**Anxiety**				**Stress**			
	***b* [CI]**	***SE B***	***t***	***p***	***b* [CI]**	***SE B***	***t***	***p***	***b* [CI]**	***SE B***	***t***	***p***
Constant	36.97 [17.57, 56.36]	9.87	3.75	0.00	34.96 [15.75, 54.18]	9.77	3.58	0.00	30.26 [10.09, 50.43]	10.26	2.95	0
Age	–0.04 [–0.35, 0.27]	0.16	–0.24	0.81	–0.14 [–0.45, 0.17]	0.16	–0.87	0.38	0.16 [–0.16, 0.49]	0.17	0.99	0.32
Gender	–0.93 [–2.31, 0.45]	0.70	–1.33	0.18	–0.91 [–2.27, 0.46]	0.70	–1.30	0.19	–1.47 [–2.91, –0.04]	0.73	–2.02	0.04
SOC	–0.55 [–0.89, –0.22]	0.17	–3.26	0.00	–0.48 [–0.81, –0.15]	0.17	–2.87	0.00	–0.5 [–0.85, –0.15]	0.18	–2.84	0
Positive epidemic information	–0.27 [–1.36, 0.83]	0.56	–0.48	0.63	–0.15 [–1.23, 0.94]	0.55	–0.27	0.79	0.13 [–1.01, 1.27]	0.58	0.23	0.82
Interaction	0.01 [–0.01, 0.03]	0.01	0.81	0.42	0.01 [–0.01, 0.03]	0.01	0.68	0.50	0.004 [–0.02, 0.02]	0.01	0.37	0.71
R^2^	0.32				0.28				0.32			
ΔR^2^	0.001				0.0008				0.0002			

*SOC, sense of coherence; b, unstandardized beta; SE B, standard error for the unstandardized beta; *t*, *t*-test statistic; *p*, *p*-value.*

**TABLE 3:** Hierarchical regression analyses of Negative epidemic information exposure on negative emotions (*N* = 424).

	**Depression**				**Anxiety**				**Stress**			
	***b* [CI]**	***SE B***	***t***	***p***	***b* [CI]**	***SE B***	***t***	***p***	***b* [CI]**	***SE B***	***t***	***p***
Constant	16.36 [3.48, 29.24]	6.55	2.50	0.01	23.61 [10.7, 36.52]	6.57	3.60	0.00	21.78 [8.15, 35.41]	6.93	3.14	0.00
Age	–0.10 [–0.41, 0.21]	0.16	–0.64	0.53	–0.19 [–0.51, 0.12]	0.16	–1.22	0.22	0.1 [–0.23, 0.43]	0.17	0.60	0.55
Gender	–0.87 [–2.23, 0.49]	0.69	–1.25	0.21	–0.81 [–2.18, 0.55]	0.69	–1.17	0.24	–1.28 [–2.73, 0.16]	0.73	–1.75	0.08
SOC	–0.15 [–0.34, 0.05]	0.10	–1.50	0.13	–0.22 [–0.42, –0.03]	0.10	–2.27	0.02	–0.26 [–0.46, –0.06]	0.10	–2.50	0.01
Negative epidemic information	1.66 [0.64, 2.68]	0.52	3.19	0.00	0.91 [–0.11, 1.93]	0.52	1.75	0.08	1.05 [–0.03, 2.13]	0.55	1.91	0.06
Interaction	–0.03 [–0.04, –0.01]	0.01	–2.74	0.01	–0.01 [–0.03, 0.01]	0.01	–1.40	0.16	–0.02 [–0.04, 0.004]	0.01	–1.59	0.11
R^2^	0.34				0.28				0.32			
ΔR^2^	0.01**				0.003				0.004			

SOC, sense of coherence; **p < 0.01; b, unstandardized beta; SE B, standard error for the unstandardized beta; *t*, *t*-test statistic; *p*, *p*-value.

In order to further explain the specific regulation of negative epidemic information exposure, participants were divided into low-exposure group (Z – 1SD) and high- exposure group (Z + 1SD) according to the standard score of negative epidemic information exposure. The simple slope test was used to investigate the effect of SOC on depression at different levels of exposure to negative epidemic information.

**FIGURE 3:**
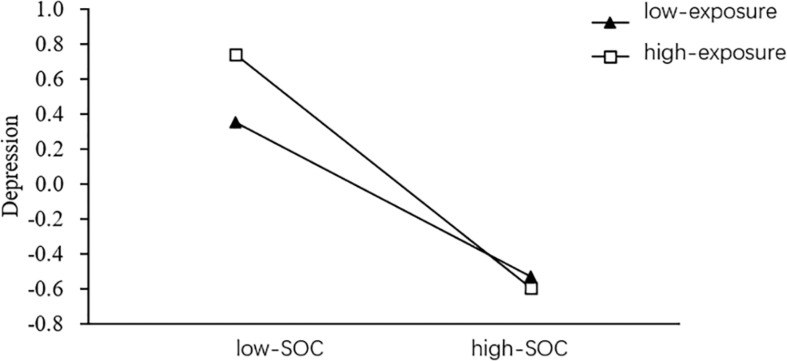
Moderated effect of negative epidemic information exposure between SOC and depression (SOC: sense of coherence; *N* = 424).

Figure 3 shows that SOC has a significant negative predictive effect on depression in low-exposure group (β = –0.45, *p* < 0.001) and high-exposure group (β = –0.67, *p* < 0.001). Compared with low-exposure, high-exposure has a higher predictive effect. It shows that with the increase of negative epidemic information exposure, the predictive effect of SOC on depression is increasing gradually.

## Discussion

This study attempted to examine the relationship between media exposure, SOC and mental health, and the moderating effect of media exposure in college students after returning to school. The results of the present study indicate that negative epidemic information exposure has a moderating effect on the relationship between SOC and depression, while positive epidemic information exposure has no significant effect. This is consistent with our research hypothesis. Consistent with the results of previous studies, our study found that the SOC was significantly correlated with college students’ depression, anxiety, and stress, and had a significant negative predictive effect on college students’ depression, anxiety, and stress (Schnyder et al., 2000; Gustavsson et al., 2007; Moksnes et al., 2013). The SOC is a controllable and meaningful self-confident tendency that an individual maintains when dealing with internal and external environmental stimuli in his or her life, reflecting one’s understanding of environmental stress and his or her ability to use existing resources to cope with difficulties, and his or her attitude to invest energy and take responsibility for difficulties (Eriksson and Lindström, 2007; Yoshida et al., 2014). In other words, individuals with a high SOC will be more likely to see it as a predictable and meaningful challenge that can be actively addressed with appropriate strategies. Therefore, individuals with a higher SOC are better at exploring and utilizing internal and external resources and adopting appropriate strategies to cope with them, so as to maintain a healthier physical and mental state and experience fewer negative emotions (such as depression, anxiety, and stress) (Gustavsson-Lilius et al., 2012).

Previous studies have shown that media reports related to crises may lead to severe psychological stress or obvious mental conditions (Holman et al., 2014; Pfefferbaum et al., 2014). Several recent studies have also found that higher levels of COVID-19 media exposure are significantly associated with greater psychological stress (Bendau et al., 2020; Yao, 2020). Media reports on specific topics caused great pressure for most people (Veer et al., 2020). Therefore, reports of COVID-19 may be a source of stress, and frequently exposure to such information may cause people to experience greater stress. In fact, previous studies have found that media exposure can prolong people’s experience of acute stress and produce substantial stress symptoms (Holman et al., 2014), and the perceived stress when experiencing negative life events is one of the main factors leading to depression (Disner et al., 2011; Li et al., 2014; Treadway et al., 2015). In the present study, information exposure was divided into positive and negative exposure. Correlation analysis showed that positive epidemic information exposure was not significantly correlated with depression, anxiety, and stress. It should be noted that female reported more positive epidemic information exposure than male. This may be because of gender difference in media choice and preference (Hou et al., 2020; Twenge and Martin, 2020). The results of this study also show that there is a significant positive correlation between negative epidemic information exposure and stress. Therefore, media exposure may influence the level of depression by influencing the individual’s perception of stress. Different from previous studies (Chao et al., 2020), our study found that negative emotions such as depression, anxiety, and stress were significantly positively correlated with exposure to negative epidemic information, but not significantly correlated with exposure to positive epidemic information. This may be due to the fact that all kinds of information at the beginning of the epidemic increased individual’s sense of uncertainty and psychological burden triggering mental health symptoms. With the epidemic under control, people can better distinguish the positive information instead of using it as a burden. When exposed to negative information about COVID-19, people may perceive more stress and have more depressive and anxiety symptoms due to the severity and harmfulness of the epidemic (Guessoum et al., 2020; Wang H. et al., 2020). Therefore, when public health events occurred, how to avoid excessive transmission of negative information in the process of correct reporting of disaster events was worth paying attention to and thinking about.

In this study, negative epidemic information exposure was positively correlated with stress, and meanwhile, negative epidemic information exposure was also positively correlated with depressive and anxiety symptoms. There was no significant correlation between positive epidemic information exposure and stress. At the same time, positive epidemic information exposure was also not significantly associated with depressive and anxiety symptoms. Studies have shown that repeated exposure to media trauma-related information may affect individuals’ assessment of threats (Marshall et al., 2007), and media reports are one of the strongest emotional stressors in the context of the current pandemic (Veer et al., 2020). In our study, no moderating role was found for the positive information exposure. At present, the COVID-19 pandemic is still spreading, and people are well-prepared for long-term anti-epidemic. The positive information exposure plays a limited soothing role, and has little impact on the risk and stress cognition of college students. However, we found that exposure of negative epidemic information plays a moderating role in the influence of SOC on depressive symptoms. That is, with the increase exposure to negative epidemic information, the negative predictive effect of SOC on depressive symptoms is gradually increasing. Under the exposure of low negative epidemic information, individuals with a high SOC can adopt appropriate strategies, while individuals with a low SOC are lack of adequate coping strategies. According to the salutogenic model, SOC determines the individual’s perception of the external environment, that is, less stress, less interference, and less chaos. Therefore, the higher the SOC, the less they experience depressive symptoms. When exposed to highly negative epidemic information, individuals with a higher SOC would selectively receive the information based on its value as a resistance resource against stressors. They could ignore the negative information (Antonovsky, 1987), avoid excessive perception of risk and stress, and thus maintain a lower level of depression. However, due to the lack of confidence in their own adaptability, individuals with low SOC are often accompanied by the impression that they are at a loss and in an out-of-control environment (Antonovsky, 1987), and a large amount of negative epidemic information is more likely to amplify their perception of risk and stress, thus increasing their level of depression. Therefore, college students’ SOC is still in the process of development. We need to take certain measures to help college students develop and enhanced their SOC, improving their ability to deal with pressure and reducing their negative emotions.

This study also has some shortcomings. First of all, this study adopted a cross-sectional design. Although this study shows that exposure to negative epidemic information plays a moderating role between psychological concordance and depressive symptoms, the cause-and-effect relationship remains unclear. Future longitudinal or quasi-experimental studies may shed light on the causal relationship between the variables. Secondly, the universality of the results of the present study may be limited by time, geography, gender, and socio-cultural background. Although gender has been controlled in data analysis as control variable in the moderation analyses, gender bias may still affect the present study results. Besides, the data of this study were collected in June 2020, and the subjects were mainly from Tianjin, Guangxi, and other places, with a small coverage area and insufficient representativeness, which may affect the generalization of the research results. Finally, self-reports may be affected by response biases, and more research, including longitudinal study design and experimental comparisons, is needed to expand our understanding of the relationship between SOC, media exposure, and depressive symptoms.

## Data Availability Statement

The raw data supporting the conclusions of this article will be made available by the authors, without undue reservation.

## Ethics Statement

The studies involving human participants were reviewed and approved by the Ethics Committee of Tianjin Normal University. The patients/participants provided their written informed consent to participate in this study.

## Author Contributions

ML, ZX, and HY designed the study and drafted and wrote the manuscript. XH, ML, JZ, and RS performed the research and acquired the data. JZ, RS, WD, and ZX interpreted and analyzed the data. ML, ZX, TL, and HY revised and replied to the reviewer’s comments.

## Conflict of Interest

The authors declare that the research was conducted in the absence of any commercial or financial relationships that could be construed as a potential conflict of interest.
